# Ongoing over-exploitation and delayed responses to environmental change highlight the urgency for action to promote vertebrate recoveries by 2030

**DOI:** 10.1098/rspb.2023.0464

**Published:** 2023-04-26

**Authors:** Richard Cornford, Fiona Spooner, Louise McRae, Andy Purvis, Robin Freeman

**Affiliations:** ^1^ Institute of Zoology, Zoological Society of London, London NW1 4RY, UK; ^2^ Department of Life Sciences, Natural History Museum, London SW7 5BD, UK; ^3^ Department of Life Sciences, Imperial College London, Ascot SL5 7PY, UK; ^4^ Our World in Data at the Global Change Data Lab, Oxford OX2 0DP, UK

**Keywords:** biodiversity change, ecological lags, Living Planet Database, population trends, vertebrates

## Abstract

To safeguard nature, we must understand the drivers of biodiversity loss. Time-delayed biodiversity responses to environmental changes (ecological lags) are often absent from models of biodiversity change, despite their well-documented existence. We quantify how lagged responses to climate and land-use change have influenced mammal and bird populations around the world, while incorporating effects of direct exploitation and conservation interventions. Ecological lag duration varies between drivers, vertebrate classes and body size groupings—e.g. lags linked to climate-change impacts are 13 years for small birds, rising to 40 years for larger species. Past warming and land conversion generally combine to predict population declines; however, such conditions are associated with population increases for small mammals. Positive effects of management (*>*+4% annually for large mammals) and protected areas (*>*+6% annually for large birds) on population trends contrast with the negative impact of exploitation (*<*−7% annually for birds), highlighting the need to promote sustainable use. Model projections suggest a future with winners (e.g. large birds) and losers (e.g. medium-sized birds), with current/recent environmental change substantially influencing abundance trends to 2050. Without urgent action, including effective conservation interventions and promoting sustainable use, ambitious targets to stop declines by 2030 may already be slipping out of reach.

## 1. Introduction

Despite international commitments to protect the natural world [1], global rates of species loss are tens to thousands of times higher than the expected background level [2] and at least one million plant and animal species are estimated to be threatened with extinction [3]. Arresting and reversing the decline in biodiversity—‘bending the curve’—requires global, concerted action [3,4].

Land-use change (LUC) is the global driver most affecting terrestrial and freshwater systems [3]. At least 70% of land has been modified by humans [5], with anthropogenic environments typically home to less diverse communities than natural habitats [6] and contributing to ecological homogenization across space [7]. Climate change (CC) is an increasingly substantial driver of ecological change [5,8], linked to bumblebee declines [9], recurrent coral bleaching [10] and the restructuring of marine communities [11]. Although currently less impactful globally than LUC and direct exploitation [3], the impacts of warming on biodiversity are expected to increase as global temperatures rise [3,12,13,14]. Many analyses treat the two drivers separately (e.g. [15,16]), or assume their effects to be additive (e.g. [13,17]). Although these approaches can offer detailed assessments of individual driver impacts, changes to climate and land use are expected to interact, with exposure to one driver influencing vulnerability to the other [18,19].

Direct exploitation is another substantial driver of biodiversity loss [3,20], yet the direct use of wildlife underpins the livelihoods of many people worldwide [21]. Ensuring the sustainability of such use is therefore critical for both people and nature. Protected areas (PAs) are an important conservation intervention, helping the maintenance and recovery of wildlife populations by reducing exposure to multiple threats, including LUC [22]. Despite this, PA coverage remains low (approx. 17% of global terrestrial area [23]), threatened species are poorly represented [24], and poor management can limit conservation success [25]. Simultaneously, PAs can restrict local peoples' access to vital natural resources [26]. Even if 30% of land is protected by 2030 [27], additional interventions mitigating exploitation [28] will be required to adequately safeguard biodiversity and nature's contribution to people.

Modelling the response of biodiversity to combinations of drivers and interventions is increasingly used to inform decision makers about the costs and benefits of conservation actions [29,30]. However, such models often use concurrent environmental and ecological data—e.g. space-for-time approaches [31] and species distribution models [32]—even though temporal delays in nature's response to pressures (lags) are often expected [33]. Changes in population abundance often lag several years behind habitat loss or degradation [34], and species that cannot sustain viable populations, but do not immediately disappear, create an extinction debt [35,36]. CC drives similar effects when species distributions are slow to track shifting climate envelopes [33,37]. Historic (1900–1910) pressures also better explain variation across Europe in the proportion of threatened species than do contemporary (2000) drivers [38].

Here, we comprehensively assess how time lags—ranging from one to 57 years—influence the response of mammal and bird population trends to both CC and LUC. In addition to statistically identifying those lags that best explain population trends, our models estimate effects of key non-environmental threats and interventions: biological resource use, PAs and targeted management. By combining these features into a single analysis, something that has not been done previously, we present a more complete picture of vertebrate abundance responses to anthropogenic actions.

Specifically, we investigate the following:

1. Do lagged effects of environmental change better explain variation in vertebrate population trends than contemporary (unlagged) effects?
2. Does expressing lags in terms of numbers of generations for each species provide a better fit than expressing lags in years?
3. Do lags differ between vertebrate classes, ecological groups (e.g. body size and trophic level) and environmental change drivers, and if so, how?
4. Do the estimated effects of environmental change vary across lags?
5. What are the ecological implications of lags on future abundance trends?

## 2. Methods

### (a) Data

Population time-series for terrestrial and freshwater birds and mammals covering 1950–2014 were obtained from the Living Planet Database (LPD; http://livingplanetindex.org/data_portal). Time-series in the LPD contain repeat measures of population size, density, abundance or a proxy for abundance [39]. We used all such data, and, for simplicity, use the term abundance to refer to all data types. For each population with a known location and a time-series spanning at least 5 years with at least three monitoring time-points, we modelled log_10_ abundance as a function of time (within the monitoring time period). Prior to modelling, zeros in a time-series were converted to 1% of the mean of non-zero entries for that time-series. In time-series with abundance values less than one, we added one to each abundance record in that time-series. We used log_10_-linear interpolation to estimate annual log_10_ abundance values for time-series with fewer than six data points. A generalized additive model (smoothing parameter set to half the number of data points) was used to predict annual log_10_ abundance values when six or more data points were available. Each population's average logged rate of annual change ($\bar{\lambda }$, a metric of relative change in abundance and subsequently referred to as ‘population trend’) was calculated as

$$\bar{\lambda }=\frac{\sum_{t=2}^{T}\log_{10}(N_{t}/N_{t-1})}{T-1},$$

where *T* is the total number of years in the interpolated time-series and *N_t_* is the abundance value in year *t* [40] (see electronic supplementary material, figure S2.15).

**Figure 1.**
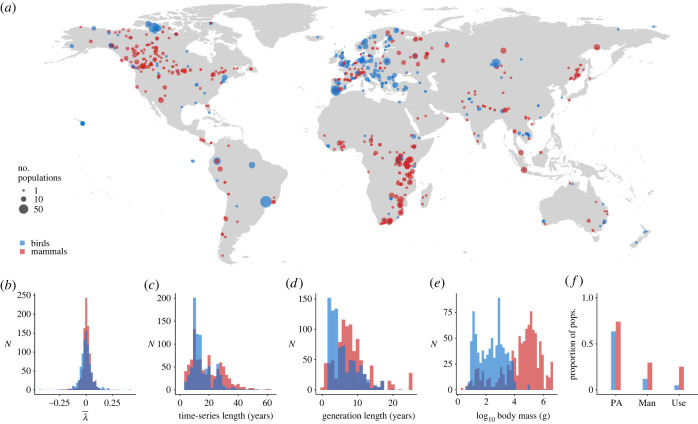
Graphical summary of data used in modelling. (*a*) Population time-series are from across the world, yet bird data are concentrated in northwest Europe, and mammals are best represented in southern Africa, western Europe and North America. Distributions of (*b*) average logged rates of population change $\left(\bar{\lambda }\right)$, (*c*) time-series length (end year − start year + 1), (*d*) generation length and (*e*) body mass. (*f*) The proportion of modelled populations that are within a PA, receive targeted management (Man) or are subject to hunting or collecting (Use).

The main analysis we present includes only populations where the trend model had adjusted *R*^2^ *>* 0, an arbitrary threshold that excludes potentially unreliable and poorly fitted time-series (removes 213 mammal populations and 293 bird populations). We also excluded two bird species (25 populations of *Gyps bengalensis* and 6 populations of *Podiceps nigricollis*) and one mammal population (species identity is confidential) that substantially influenced model coefficients (Cook's *D* *>* 0.5; electronic supplementary material, table S1.1). One of the excluded species—white-rumped vultures (*Gyps bengalensis*)—has severely declined since 1990 due to diclofenac poisoning [41], a highly specific situation that is not representative of the general patterns in the wider dataset. Our main analysis is therefore based on 1751 populations, of 712 species across 664 locations (figure 1; electronic supplementary material, table S3.2).

Temperature and human land-use data spanning 1901–2100 were collated from the IPSL-CM6a-LR [42] and Land-Use Harmonization 2 (LUH2) [43] databases, respectively. Average daily temperature was extracted from each 0.5^◦^ IPSL grid cell containing any of the populations. Each population's experienced rate of CC (without lags) was estimated by the slope of a linear model of mean annual temperature against time across the monitoring period [40]. Likewise, we extracted annual proportions of anthropogenic land cover (i.e. cropland plus pasture plus rangeland) from each population's 0.25^◦^ LUH2 grid cell, and estimated rates of change in land use as the mean of annual differences over the monitoring period [40]. For details on calculating lag-adjusted rates, see the Lags section of the Methods. Although a number of global environmental datasets are available, we focus on IPSL and LUH2 due to their broad temporal extent and use in recent intercomparisons of global biodiversity models [30,44]. In additional analysis, we also used data from CRU 4.04 [45] (temperature) and HYDE 3.2 [46] (land use); this sensitivity analysis is reported in the electronic supplementary material: Sensitivity to environmental data sources. Further analysis of how alternative land-use datasets may influence model outputs can be found in electronic supplementary material: Comparing land-use datasets.

### (b) Model structures

Broadly following Spooner *et al.* [40], we fitted linear mixed-effects models [47] linking population trends ($\bar{\lambda }$) to (lagged) rates of CC and LUC, log_10_ body mass (BM; sourced from the Amniote database [48]) and PA status (PA, see model 1, Base, table 1).

**Table 1.** Summary of fixed effect model structures considered.

model	fixed effects
1 Base	CC*LUC + BM + PA
2 +MU	CC*LUC + BM + PA + Man + Use
3 +R	CC*LUC + BM + PA + Realm
4 +MUR	CC*LUC + BM + PA + Man + Use + Realm
5 Null	N/A

All models included species and location as random intercepts.

CC = rate of climate change, LUC = rate of land-use change, BM = log_10_ body mass, PA = protected area status, Man = management status, Use = biological resource use status and Realm = biogeographic realm. CC and LUC were estimated based on ecological lags; see Methods: Lags.

Building upon this model structure, we also fitted models including categorical, fixed effects for the biological resource use (Use; e.g. hunting and collecting) and management (Man; e.g. legal protection and harvest quotas) status of populations (model 2, +MU, table 1). These data fields are recorded in the LPD, having been obtained from population data sources (see electronic supplementary material, table S3.3, and [28] for details).

We additionally considered biogeographic realm (Realm) [49] as a fixed effect to account for variation in population trends linked to differing environmental change histories [50] and species pools [51]. Models in which Realm interacted with CC*LUC, PA, Man and Use were over-parameterized (rank-deficient, lack of available covariate combinations to estimate all fixed effects) for birds, so our main analysis treats Realm as an additive effect only (models 3, +R, and 4, +MUR, table 1).

All continuous, explanatory variables (CC, LUC and BM) were centred and scaled prior to model fitting. Random intercepts for species identity and population location were included to account for taxonomic and spatial variation in population responses. Due to their different ecological characteristics, birds and mammals were modelled separately. We also compared the above model structures with a null model for each class (i.e. a global intercept with random intercepts for species identity and population location).

### (c) Lags

We define an ecological lag (lag) as the time delay between environmental changes (here, related to temperature and land use) and the biodiversity response that they drive (population trend). For model fitting, we considered time lags in two currencies: years and generations. Given the temporal limits of the abundance (1950) and environmental (1901) data, we considered year-based lags of between 0 and 49 years. Using generation length data ([52] for mammals and [53] for birds), we also modelled species-specific generation-based lags within the range 0.3–3.1 generations for birds and 0.3–2.3 generations for mammals. Again, these limits were determined by the temporal extent of the environmental data. We converted these generation-based multipliers to whole years for modelling (see below). For example, a lag of two generations corresponds to 50 years for African elephants (2 × 25 years), and 2 years for Eurasian pygmy shrews (2 × 1 year).

We used these lags to temporally offset the start of environmental time-series from the start of each population time-series, using the same lag for all populations (e.g. two generations). For each of the year- and generation-based approaches, we considered all possible combinations of climate and land-use lags (e.g. when specifying a 5-year lag for CC, we considered lags of 0–49 for LUC). We therefore evaluated 3341 lag combinations for birds (50^2^ year-based + 29^2^ generation-based) and 2941 for mammals (50^2^ + 21^2^). Environmental time-series were set to be equal in length to the associated population time-series and rates of change (CC and LUC) were calculated using the procedure outlined above (electronic supplementary material, figure S2.16). These lagged rates of change were used in model fitting.

### (d) Ecological subsetting

While generation length measures one aspect of a species' life history, other ecological traits are also expected to influence ecological lag duration [54]. We therefore separately evaluated how optimal lags varied depending on body mass, trophic level and latitude. We investigated these ideas by subsetting the mammal and bird populations based on the above-described features and fitting all lag-based models described previously. For body mass, we split the data into three equal parts (using tertiles), generating subsets for small, medium and large species. Our trophic level split divided species into carnivores (diets contain at least 2/3 animals) and herbivores (diets contain at least 2/3 plants; diet data from [55]). Finally, we split data to temperate (above 23.5° N or below −23.5° S) and tropical (below 23.5° N and above −23.5° S) populations.

### (e) Model evaluation

We ranked models (and lags) using AICc (Akaike's information criterion corrected for small sample size) and Akaike weights [56]. Akaike weights (sum to 1) indicate the relative likelihood of a model given the data (i.e. support relative to other models considered) and provide a basis for model averaging [56]. For each vertebrate class, model structure and ecological subset combination, we retained the most plausible set of models/lags (ΔAICc *<* 6 [57]), averaged these models based on Akaike weights, and investigated the effects of the modelled drivers and conservation approaches.

### (f) Investigating the consequences of lags

Using the retained models (ΔAICc *<* 6) and their associated lags, we projected an index of relative abundance from 2010 onwards for populations in our model-fitting data. While these projections are therefore not necessarily representative of abundance change for all vertebrates (or even all birds and mammals), they offer useful insight into the potential impacts of lags and alternative development pathways. We used temperature and land-cover values, for each population location, from three socio-economic scenarios (SSP1 RCP2.6, SSP3 RCP7.0 and SSP5 RCP8.5), representing different possible environmental futures. SSP1 RCP2.6 offers a ‘sustainable’ future with low land-use pressure and limited CC [58]. SSP3 RCP7.0 represents a ‘regional rivalry’ scenario, with moderate–high climate warming and large expansion of cropland and pasture [59]. SSP5 RCP8.5 is an energy- and resource-intensive scenario with moderate land-use pressure and high CC [60]. Lagged rates of environmental change were calculated for each population, scenario, decade, and model combination (e.g. Population 1, SSP1 RCP2.6, 2010–2020, Model A [ΔAICc = 0]). We assumed PA, Management and Use status remain as recorded in the LPD. Combining these variables with our retained models, we predicted average annual abundance trends per population *p* (species *s*, realm *r*, class *c*), scenario *x*, decade *d* and model $m\;({\hat{\bar{\lambda }}}_{\;p,s,r,c,x,d,m})$. We then hierarchically averaged these predictions, first to ${\hat{\bar{\lambda }}}_{s,r,c,x,d,m}$, then ${\hat{\bar{\lambda }}}_{r,c,x,d,m}\;$ and then ${\hat{\bar{\lambda }}}_{c,x,d,m}$. Model-averaged values $\left({\hat{\bar{\lambda }}}_{c,x,d}\right)$ were then calculated based on Akaike weights. We converted these average predicted trends to an index value $\left(I_{c,x,d}\right)$ as: $I_{c,x,d}=I_{c,x,d-1}\cdot 10^{\left(10\cdot {\hat{\bar{\lambda }}}_{c,x,d}\right)}$; where $I_{c,x,d-1}$ is the index value from the end of the previous decade and ${\hat{\bar{\lambda }}}_{c,x,d}$ the average rate of change over decade *d* [61,62]. Index values for 2010 were set to 1.

## 3. Results

### (a) Lag type and model structure

We found strong support for ecological lags (electronic supplementary material, figure S2.17), but species-specific generation-based lags were not clearly better than simpler year-based versions (electronic supplementary material, figure S1.1). For birds, the best model structure included management and use (+MU; electronic supplementary material, figure S2.17). Although the model structure including management, use and Realm (+MUR) has the lowest AICc score for mammals, a model without Realm (+MU) has a ΔAICc of 2.2, suggesting the simpler models are also well supported [57]. For simplicity and comparability of parameters between birds and mammals, we therefore present all subsequent results based on model structure + MU for both groups (results based on + MUR models can be found in electronic supplementary material, figure S1.3 and table S3.5).

### (b) Optimal lag duration

**Figure 2.**
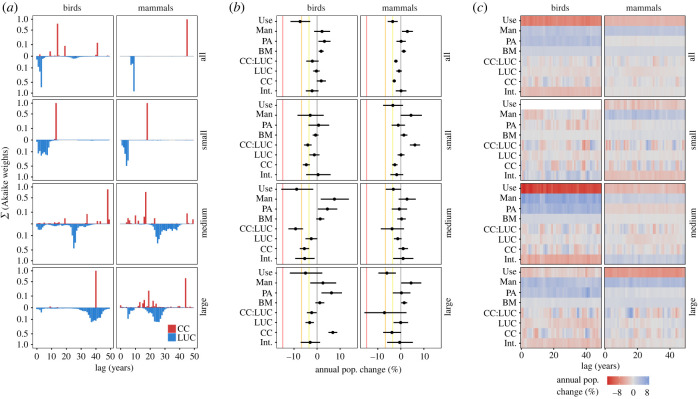
Support for ecological lags (*a*), model-averaged coefficients (*b*) and coefficient variation across lags (*c*). (*a*) Models incorporating lags are an improvement upon those without. The best-supported lags vary between classes and body size groupings, but larger species tend to display longer ecological lags than do smaller species. While the optimal lags linked to small birds are 13 years for CC and under 10 years for LUC, these rise to 40 years for large birds. (*b*) The model-averaged coefficients suggest complex effects of environmental change—both positive and negative effects of CC and CC:LUC. A clear negative impact of use on population trends is also estimated for both vertebrate classes, with consequences generally equivalent to IUCN Red List categorization of endangered (yellow line) and vulnerable (orange line), for birds and mammals, respectively. By contrast, management (Man) is associated with more positive population trends, with PAs appearing to benefit only birds. (*c*) Coefficients not directly linked to lagged variables (BM, PA, Man and Use) remain fairly consistent across lags and body mass subsets. The effects of CC and LUC are, however, much more variable with either positive or negative impacts being inferred depending on the lag considered. In (*a*), summed Akaike weights depict a measure of relative support for a particular lag, weights close to one indicate strong support. Values are presented on a square root scale to enhance visualization of the range of lags with relatively low support. In (*b*), coloured lines correspond to IUCN Red List threat categories based on population declines of 30% (yellow; Vulnerable), 50% (orange; Endangered) or 80% (red; Critically endangered) over 10 years (A2 criteria). In (*c*), coefficients are shown for models where both climate and land use have the same lag.

When considering models containing all populations, the optimal environmental lags differ between vertebrate classes and drivers (figure 2*a*, all; electronic supplementary material, table S3.4). Lags linked to LUC are consistently shorter than 10 years (3 years for birds and 9 years for mammals). By contrast, lags associated with climate warming are longer: 14 years for birds (although there is some support for 41-year lags) and 45 years for mammals.

Although species-specific generation-based lags do not clearly outperform simpler year-based versions, we find lags vary across body mass (figure 2*a*), and other ecological (electronic supplementary material, figure S1.5a) groupings. We include the results of body mass splits in the main text as these models demonstrate similar or higher *R*^2^ than the models for all populations (electronic supplementary material, tables S3.4 and S3.6–S3.8), while also revealing additional, interesting ecological phenomena (results based on trophic level and latitudinal subsetting can be found in electronic supplementary material: Additional ecological subsetting of population data). Generally, larger species display longer ecological lags than smaller species. For example, climate-associated lags of 13 years best explain the population trends of small birds, while this value is 40 years for large birds. Similar patterns are seen for lags associated with land use, and in mammals (figure 2*a*).

### (c) Effects of drivers and interventions

We find that biological resource use is generally associated with large, negative impacts on population trends (figure 2*b*). For birds (all), exploitation is associated with population declines of a magnitude that would warrant classification as Endangered by the IUCN (A2 criterion, *>* 50% decline over 10 years). The less negative effect on mammals (all) would correspond with a classification of Vulnerable (A2 criterion, *>* 30% decline over 10 years). By contrast, targeted population management is associated with more positive population trends (figure 2*b*, Man). In the case of large mammals, management is associated with increasing population trends by over four percentage points per year. PAs separately offer benefits to most birds (ranging from less than 1% per year for small species, to greater than 6% per year for large ones). The effects of PAs, management, biological resource use and body mass remain fairly consistent across possible lags (figure 2*c*, solid bands of colour apart from small birds).

The coefficients associated with CC and LUC are, however, more variable, across both ecological groups (figure 2*b*; electronic supplementary material, figure S1.5b) and lags (figure 2*c*; electronic supplementary material, figure S1.5c). The delayed impact of warming is estimated to be positive in some groups (all birds, large birds and medium mammals), negative in the remainder, and highly variable across lags (figure 2*c*). LUC has more neutral impacts for mammals, but negative effects for medium–large birds. Again, these estimates vary over lags, but in a much smoother pattern than seen for CC, likely reflecting the lower inter-annual variability in LUH2 anthropogenic land-use classes compared to average annual temperatures in IPSL (see electronic supplementary material, figure S2.16, for an example). The interaction between CC and LUC (CC:LUC) is negative in all body mass groups apart from small mammals (figure 2*b*), indicating that higher rates of change in one environmental variable (e.g. faster warming) are associated with a more negative relationship (slope) between the other environmental variable and population trend responses. However, as with CC coefficients, estimated interaction terms vary substantially over lags (figure 2*c*).

### (d) Ecological implications

**Figure 3.**
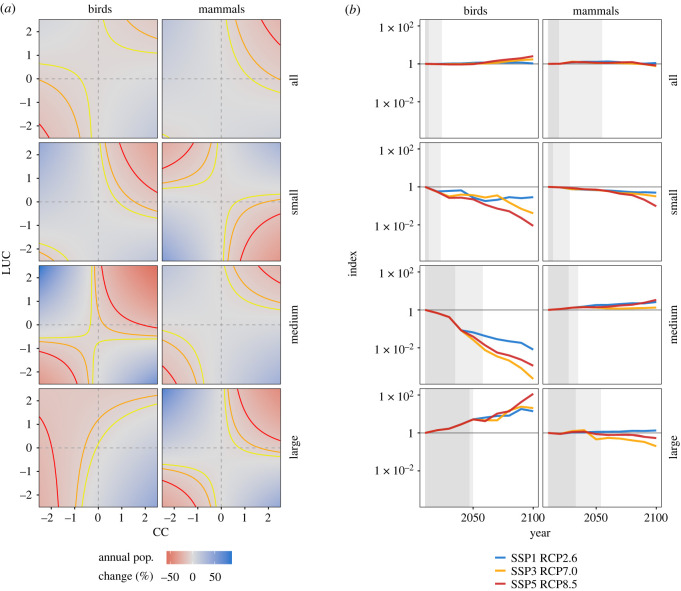
Complex, delayed impacts of environmental change on mammal and bird abundance trends. (*a*) Predicted population trends in response to combinations of scaled environmental change. While warming and land conversion (upper right of each panel) are generally associated with strong population declines, small mammals might benefit from such conditions. (*b*) Projected indices of relative abundance, based on modelled populations and alternative socio-economic scenarios, highlight important consequences of ecological lags. First, population trends up to 2050, both increases and declines, may already be largely ‘locked-in’ (grey shading) due to their dependence on environmental change that has already occurred. Second, lags of 30 years or more mean that current environmental change will substantially affect abundance trends in 2050 and beyond. In (*a*), coloured lines correspond to IUCN Red List threat categories based on population declines of 30% (yellow; Vulnerable), 50% (orange; Endangered) or 80% (red; Critically endangered) over 10 years (A2 criteria). In (*b*), the horizontal line is set at 1, the baseline for our projections. Shaded areas show future projections that are fully (dark) or partially (light) dependant on environmental change prior to 2010 (in all but medium mammals, CC is associated with longer lags and thus the lighter shading). The *y*-axes in each panel of (*b*) are kept constant to emphasize the variability between projections for different size classes. See electronic supplementary material, figure S2.18, for panels with their own scales.

The complex, delayed effects of CC and LUC on population trends are further highlighted in the prediction surfaces of figure 3*a*. In most cases, the combined effects of warming and agricultural expansion are negative for mammal and bird populations (upper right corners of panels in figure 3*a*). However, these conditions are associated with more positive population trends for small mammals. At the same time, positive population trends are generally predicted where one environmental variable increases (e.g. warming, positive CC) as the other decreases (e.g. reduction in agricultural land, negative LUC). Categorizing these combined effects of CC and LUC as either synergistic or antagonistic [63] reveals a mixed picture (electronic supplementary material, figure S1.6b). Strong positive and negative population trends appear linked to synergistic interactions between the environmental change drivers. Yet, antagonistic effects are also important, especially for large birds facing positive LUC.

Projecting models into the future suggests that aggregated indices of predicted population change may vary between extremely positive (e.g. large birds) and extremely negative (e.g. small birds; figure 3*b*). In both cases, ecological lags mean that up to 2050, population abundance will still be responding to environmental change that happened before 2010 (shaded areas in figure 3*b*). While not an issue for populations/groups that are increasing (e.g. large birds and medium mammals), such lags could hamper current conservation efforts for declining populations, such as small/medium birds and small mammals. Crucially, lags of 30 years or more, as found here for medium/large species, also mean that population trends in 2050 and beyond will be highly influenced by current environmental change and the policy decisions we make now. Our models indicate that the most sustainable future scenario (SSP1 RCP2.6) is typically associated with the most positive abundance trends (except for large and all birds, and medium mammals).

## 4. Discussion

How urgently do we need to act to meet biodiversity targets for 2030 and 2050 such as those that have been agreed at COP 15 (15th Conference of the Parties to the Convention on Biological Diversity)? The most comprehensive analysis to date of projections from multiple global biodiversity models [4] suggested that many indicators could stop falling—and even start to increase—by 2050, if concerted and ambitious actions are taken at scale. However, none of the models considered allowed for ecological time lags between environmental and biodiversity change [35], nor the impacts of CC and biological resource use. Here, using data for birds and mammals from around the world, we show that population trends are best explained by past changes in temperature and anthropogenic land use, with direct (over-)exploitation also an important (but immediate) driver of declines. Model projections indicate that both increases and declines are expected for future bird and mammal abundance, with populations up to 2050 still responding to environmental changes that have already happened. Even radical land restoration efforts [4] may therefore fail to end population declines by 2030 [64]. Additional and immediate action is needed to ensure ambitious targets for biodiversity recovery are met.

Biodiversity change in the Anthropocene is complex [7,65,66,67], as is its relationship with environmental change [11,19,34]. Delayed responses to anthropogenic development [38], habitat loss/fragmentation [68,69] and climate warming [37,70] occur across a range of taxa. Previous research had already shown lagged effects of habitat change on vertebrate populations [34] and communities [54,71]. Here, we have shown that delayed impacts of both land conversion and CC best explain vertebrate abundance trends. Although Daskalova *et al.* [34] found that lags linked to forest loss correlated with generation length, we found no benefit to including species-specific generation-based lags over year-based alternatives. We did, however, show that larger species typically display longer ecological lags than smaller ones, while patterns in the lags associated with different trophic levels and latitudes are less clear. Future studies investigating how population responses are affected by other life-history/ecological traits, and lags, will further improve our understanding of biodiversity change [72]. Our results also suggest that climate warming is linked to longer lags than LUC, possibly due to the driver's more gradual pace of change and broader/coarser geographical scale [13]. Generally, the lagged effects of fast warming, combined with high rates of land conversion are associated with declines for both vertebrate classes, due to a substantial negative interaction between the two drivers. However, this pattern is reversed for small mammals (i.e. increases are linked to warming and land conversion), due to a strong positive interaction term. With the cumulative consequences of these stressors yet to be fully realized, most current models, as well as global syntheses, such as the IPBES Global Assessment [3], might therefore underestimate the importance of LUC and CC as drivers of biodiversity loss [54].

We have shown that models incorporating lags are a substantial improvement on those that use concurrent environmental data. Yet, the estimated effects of environmental change on vertebrate abundance vary depending upon the lags considered (figure 2*c*), again highlighting the complexity of (modelling) biodiversity change. We therefore recognize that future models, more flexibly incorporating continuous, historical time-series for multiple stressors, are likely to more accurately capture the real-world dynamics of ecological responses to environmental change [18,54,73]. Furthermore, while we focus on relationships between rates of change, population responses also depend on the alignment between species' niche limits and environmental conditions [74,75]. For example, extreme temperature events—relative to a species’ niche—can cause immediate mortality [76] and may therefore be linked to shorter lags than average temperature changes. Including such species-specific contextualization of drivers therefore represents a useful avenue for further improving model performance.

In addition to the importance of lagged responses to environmental change, we find exploitation of vertebrate populations to be a substantial and immediate threat. Recent analysis suggests that hunting is the predominant threat to birds and mammals globally [20], and populations subject to biological resource use are declining more than those that are not [28]. Socio-economic factors are increasingly recognized as strongly influencing wildlife population trends [77,78], and these drivers need to be addressed alongside climate warming and habitat loss to promote the recovery of biodiversity.

Effective conservation interventions are increasingly being identified (e.g. the Conservation Evidence project; https://www.conservationevidence.com). Targeted management, such as rigorously implemented hunting quotas, could rapidly benefit wildlife populations [28], in turn buffering against declines due to other factors. However, species-specific interventions, e.g. control of invasive species and re-introduction, can be expensive and labour intensive, limiting their breadth of application. PAs therefore represent a complementary, and more general, conservation approach. Although, like Spooner *et al.* [40], we do not find PAs to be effective at promoting mammal population growth, most mammal populations analysed are within PAs (74%), making potential benefits difficult to detect. However, we do identify a strong benefit for birds, and further expansion of, and investment in, PAs could provide a critical contribution to biodiversity conservation by shielding species and ecosystems from a range of threats [25,27,79]. The positive influence of PAs is typically achieved through limiting habitat degradation [80], highlighting the benefits of restoring land to natural/semi-natural states. Restored landscapes not only help to conserve biodiversity, but the ecosystem services they provide, such as carbon sequestration, also benefit people [81]. Finally, only prompt coordinated measures to reduce carbon emissions can minimize warming [82] and minimize both immediate [12,13]—and delayed—declines in taxa vulnerable to CC.

There is wide recognition that time is short for the integrated, ambitious actions needed to stop biodiversity loss by 2050 (e.g. [3,4,83]). This work shows that time is even shorter than had been thought. On top of possible time lags between policy decision and practical action [84], our analysis suggests time lags between even immediate action and its effects on vertebrate populations. Abundance trends up to 2050 may already be largely ‘locked in’ due to their dependence on LUC and climate warming that has already occurred. While some populations are expected to increase, many are not. Ambitious targets to promote biodiversity recovery by 2030 [64,85] may already be slipping out of reach.

## Data Availability

Code and data associated with this work can be found at https://github.com/rcornf/lpi_lags_2023 and https://doi.org/10.5281/zenodo.7745401 [86]. Details and results associated with additional analyses are provided in the electronic supplementary material [87].
